# Drug-Eluting Bead, Irinotecan (DEBIRI) Therapy for Refractory Colorectal Liver Metastasis: A Systematic Review

**DOI:** 10.7759/cureus.50072

**Published:** 2023-12-06

**Authors:** Faiza H Soomro, Hafsa A Osman, Merna Haridi, Natalie A Gonzalez, Sana M Dayo, Umaima Fatima, Aaiyat Sheikh, Chaitanya S Puvvada, Ann Kashmer Yu

**Affiliations:** 1 General Surgery, California Institute of Behavioral Neurosciences & Psychology, Fairfield, USA; 2 General Surgery, Ninewells Hospital, NHS Tayside, Dundee, GBR; 3 Pediatrics, California Institute of Behavioral Neurosciences & Psychology, Fairfield, USA; 4 Medical Education, St. Martinus University, Willemstad, CUW; 5 Medical Education, California Institute of Behavioral Neurosciences & Psychology, Fairfield, USA; 6 Public Health Sciences, Liaquat University of Medical and Health Sciences, Jamshoro, PAK; 7 Obstetrics and Gynaecology, California Institute of Behavioral Neurosciences & Psychology, Fairfield, USA; 8 Internal Medicine, California Institute of Behavioral Neurosciences & Psychology, Fairfield, USA; 9 Internal Medicine, Era's Lucknow Medical College, Lucknow, IND; 10 General Surgery, Gayatri Vidya Parishad Institute of Health Care and Medical Technology, Visakhapatnam, IND

**Keywords:** debiri, drug-eluting beads, chemoembolisation, liver resection, irinotecan, colorectal cancer

## Abstract

Colorectal cancer and related mortality present a profound challenge in its management, even in this modern age. Even today, colorectal cancer-related deaths rank third in the world. Despite having multiple lines of chemotherapy, combined with radiotherapy and chemoembolization techniques, after or before surgical resection, the five-year survival rate is approximately 20%. Drug-eluting bead, irinotecan (DEBIRI) is a new technique that involves embolization of the feeding vessels to the tumour and delivering irinotecan for its chemotherapeutic effects. A significant amount of literature compares DEBIRI as an adjunct to various lines of chemotherapy. However, so far, not much data are available on DEBIRI as a singular treatment for those patients who have had multiple chemotherapies and still progressing and are not fit for liver resection. In this systematic review, we aim to highlight and bring together the results of those studies that focused on this specific patient group. A systematic search of the literature involving three large databases (published between January 2017 and July 2022), excluding languages other than English, was conducted to identify articles documenting patients who had disease progression despite chemotherapy and were not fit for surgical resection. The level of evidence and the quality check were assessed by two independent reviewers, and consensus with the senior author resolved disagreements. Out of seven studies that met the final criteria, we found a pooled cohort of 302 patients. The mean age of the patients was 61.2 years, ranging from 40.7 to 84 years. The most commonly used DEBIRI beads were M1 (70-150 um) and M2 (100-300 um), but two studies reported the use of 40 um as well. The total number of DEBIRI treatments performed in our pooled cohort was 904. The majority of the studies reported only G1/G2 toxicities among the patients, with maximal toxicity of G4 in a few selected patients. The median overall survival in our pooled cohort was 19.52 months. The median progression-free survival in our data was 5.76 months. Our systematic review concludes that DEBIRI is undoubtedly a useful treatment modality with an acceptable toxicity profile. This treatment offers a good overall survival benefit for refractory colorectal liver metastasis.

## Introduction and background

With a projected 881,000 deaths in 2018, colorectal cancer has the second most common mortality and ranks third most common cancer globally [1]. In 2020, the World Health Organization's Global Cancer Observatory reported that more than 1.9 million new cases of colorectal cancer (anal cancer inclusive) were diagnosed, and 935,000 people died from the disease, accounting for around one in 10 cancer diagnoses and deaths [2]. In addition, 60% of these individuals will develop liver metastases, making the liver the second-most common location of distant spread after lymph nodes and the site of refractory progression [3]. Within five years of an initial colorectal cancer diagnosis, one in four patients will develop liver metastases and 30-50% of patients may be diagnosed with liver progression at some point throughout their disease [4]. Although the five-year survival rate for individuals with liver metastases from colorectal cancer is improving, it is still only around 19.2% [5].

Liver surgery and liver-directed therapies have evolved to improve these survival rates and are becoming more complex and modern, incorporating minimally invasive techniques and newer treatment regimens. Despite the fact that surgical resection can offer a cure, not all patients are fit for surgery, either due to tumour size, disease extent, or insufficient hepatic functional reserve [6]. At first presentation, only 15-30% are surgically resectable [7], and the vast majority of patients with colorectal liver metastases are unresectable and are treated with systemic chemotherapy or in combination with loco-regional therapies, including transarterial chemotherapy, radioembolization, radiofrequency ablation (RFA), and microwave ablation (MWA) [8]. For those not candidates for surgery, systemic chemotherapy is used as the initial management strategy for unresectable metastatic colorectal cancer [9]. The advanced chemotherapy regimens FOLFOX (leucovorin, 5-fluorouracil, and oxaliplatin-based therapy) and FOLFIRI (leucovorin, 5-fluorouracil, and irinotecan-based therapy), as well as targeted therapies, have revolutionized the treatment of colorectal liver metastases, improving the patient's survival dramatically and prolonging median survival to up to 30 months [10,11]. However, response rates might drop to as low as 12% when a patient has not responded to first-line or, in certain situations, second-line chemotherapy [9].

In the early 20th century, drug-eluting beads (DC Beads) were used for intra-arterial delivery of irinotecan to treat colorectal cancer liver metastases [12]. Drug-eluting bead, irinotecan (DEBIRI) is a consolidative therapy designed to treat colorectal liver metastases by using transarterial chemoembolization (TACE); the primary objective is to embolize the arteries feeding the tumour site, thereby depleting oxygen and nutrients. The tangential aim is to deliver irinotecan in a well-regulated fashion directly to the tumour site. These combined effects significantly enhance irinotecan's cytotoxicity to the liver lesion and potentially reduce systemic toxicity compared to intravenous chemotherapy [13]. Irinotecan inhibits topoisomerase II, a semi-synthetic analogue of the naturally occurring alkaloid camptothecin. In phase II studies, irinotecan demonstrated activity in fluorouracil resistant with metastatic colorectal cancer; response rates of 11-23% have been achieved for this drug in such cases [14,15]. Chemoembolization, which uses drug-eluting beads to deliver chemotherapy directly into the blood supply of tumours, was developed as a way to improve the pharmacokinetic profile of the chemotherapeutic agents being delivered [16] and the drug-eluting beads could be loaded with irinotecan, as it has shown to have single-agent efficacy in the treatment of colorectal cancer [6]. In the pretreated population of patients with unresectable colorectal liver metastases, DEBIRI showed an intention-to-treat overall response rate of 70%, and tumour control lasted for 15 months [13].

The effectiveness of DEBIRI therapy and its relatively low toxicity profile make it a favourable local-regional treatment option for chemotherapy-refractory colorectal liver metastases [17]. This systematic review will assess the best available evidence and establish the cumulative safety and efficacy profile of DEBIRI for patients with unresectable colorectal liver metastases.

## Review

Methods

**Figure 1:**
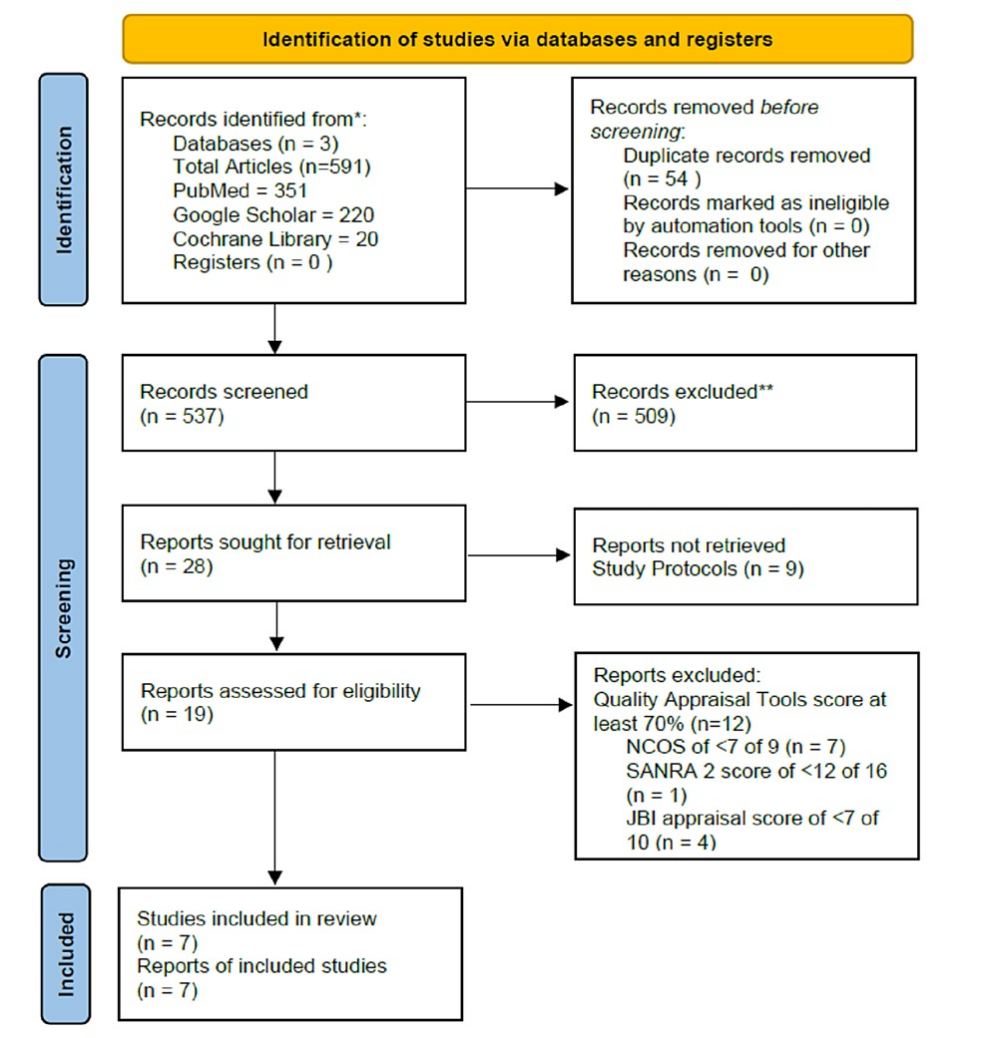
Flow chart of the study search selection NOSC: Newcastle-Ottawa Scale; SANRA 2: Scale for the Assessment of Narrative Review Articles 2; JBI: Joanna Briggs Institute critical appraisal tools.

This systematic review was conducted against the Preferred Reporting Items for Systematic Reviews and Meta-Analyses (PRISMA) 2020 guidelines [18]. Figure 1 summarizes the literature search and selection methodology.

Literature Search

A structured search was performed in the PubMed/MEDLINE database, Cochrane Collaboration Library, and Google Scholar for studies that were published in the last five years (2017-2022). The keywords used were DEBIRI, irinotecan, colorectal liver metastasis, and survival benefit. We limited our results to the English language in human subjects. References of critical articles were also cross-checked. We identified manuscripts evaluating overall survival benefits after DEBIRI treatment in patients with colorectal liver metastasis, neither responding to conventional chemotherapy nor amenable to surgical resection. The last date of our search for articles was 14/07/2022.

As shown in Table 1, the exclusion criteria for the studies included DEBIRI as a singular treatment and its results in patients with colorectal liver metastases who have been deemed unfit for surgery, patients who underwent multiple lines of conventional chemotherapy but still showed disease progression, articles not in English language, and studies on animal subjects. Studies where DEBIRI was paired with any other loco-regional therapy (radiofrequency ablation, microwave ablation, selective internal radiation therapy, transarterial chemoembolization), any other form of intra-arterial therapy, or any other chemotherapy (e.g., DEBIRI with FOLFOX/FOLFIRI, or DEBIRI combined with capecitabine) were also excluded. Studies with smaller sample sizes (less than five) were also excluded. Other reasons for exclusion included case reports, editorials, letters to the editor, and literature reviews. The focal study characteristics have been described in Table 2.

**Table 1:** Inclusion and exclusion criteria for search strategy CRLM: colorectal liver metastases; DEBIRI: drug-eluting bead with irinotecan.

Inclusion criteria	Exclusion criteria
Treatment modality assessed is only DEBIRI	DEBIRI paired with loco-regional therapy
Patients included in studies had progression of disease despite having conventional chemotherapy	DEBIRI compared with other forms of chemotherapy
CRLM does not meet the criteria for surgical resection	Sample size less than 5
Articles in English	Types of studies: editorials, review articles, case reports
	Animal studies

**Table 2:** Study characteristics DEBIRI: drug-eluting beads loaded with irinotecan; mCRLM: metachronous colorectal liver metastasis; CRLM: colorectal liver metastasis; DEBs: drug-eluting beads; CRCLM: colorectal cancer liver metastasis; VEGF: vascular endothelial growth factor; VEGFR1: vascular endothelial growth factor receptor 1.

Reference	Sample size (N)	Conclusion	Level of evidence
Mauri et al. June 2018 [19]	18	Small beads (70–150 μm) or larger beads (100–300 μm) show no difference in outcomes; however, more toxicity was observed in treatments with smaller beads. Both groups showed a triad of pain hypothermia and high blood pressure named as DEBIRI-specific post-embolization triad. It is essential for the treating speciality to be well aware of this syndrome for appropriate management.	4
Scevola et al. February 2017 [20]	62	DEBIRI of mCRLM in patients with metastases dominant in the liver appeared to be an effective treatment. It is well tolerated with more safety profile. The survival rate was increased in patients who did not respond to second-line chemotherapy previously.	3
Ngo et al. May 2019 [21]	53	Median survival = 14.5 months. Median hepatic progression-free survival = 5 months. Of the population, 45% show extra-hepatic disease, and it is rendered as a poor prognostic factor for overall survival. If the patient has received previous ablation and systemic chemotherapy, overall survival was prolonged.	3
Mauri et al. January 2022 [22]	55	Small bead DEBIRI embolization treatment is a safe and effective procedure. It is a salvage treatment for CRLM patients, showing dependable results and prolonged survival outcomes.	3
Kong et al. June 2019 [23]	16	CalliSpheres DEBs is a secure treatment approach for CRCLM, but it results in a high local response rate. Hypoxia caused by embolization induces increased production of vascular endothelial growth factor (VEGF), stimulating angiogenesis. This promotes tumour recurrence. DEBIRI, in combination with angiogenesis inhibitors, may be studied in the future.	4
Fereydooni et al. October 2018 [24]	14	The use of DEBIRI in the treatment-refractory population has acceptable pharmacokinetics. It is technically attainable with high success rates. Patients with liver-dominant disease can tolerate it well. VEGFR1 is a predictor of treatment efficacy and risk of adverse events. VEGFR1 levels significantly reduced at 24 hours.	2
Boeken et al. February 2020 [25]	84	No difference in outcomes was discerned between DEBIRI bead sizes (70–150 μm versus 100–300 μm). Smaller beads showed higher toxicity after treatments.	2

Data Extraction

Two reviewers independently appraised each article to extract data for prearranged study variables (Table 3). After a thorough search of the three databases, a total of 591 citations were retrieved. Until the final selection of the articles included in our systematic review, the citations underwent duplicate removal and exclusion based on the titles and abstracts. Titles that seemed appropriate or the studies that could not be excluded unambiguously from the title and abstract were identified, and two reviewers reviewed the corresponding full-text reports. Any disagreement was resolved by consensus with the senior author.

**Table 3:** Demographics and clinical characteristics of the study population ECOG: Eastern Cooperative Oncology Group; CEA: carcinoembryonic antigen; PS: performance status.

Study		Age	Male	Female	ECOG performance status			CEA
					PS 0	PS 1	PS 2	
Mauri et al. [19]		61.2 (46–81)	11 (61.1%)	7 (38.9%)	18/18 (100%)	-	-	253.6 (10.5–810.6)
Scevola et al. [20]		61.7 (range: 47–75)	39	23	NR	NR	NR	113 (range: 21.2–1376)
Ngo et al. [21]		71 (41–88)	39 (74%)	14 (26%)	NR	NR	NR	NR
Mauri et al. [22]		64.5 (40.7–82.9)	32 (58%)	23 (42%)	36 (65%)	17 (31%)	2 (4%)	298.5 ± 506
Kong et al. [23]		54.6 (45-68)	10	6	14 (87.5)	2 (12.5)	0 (0%)	194.92 ± 58.80
Fereydooni et al. [24]		52.5 (44–84)	6	8	8 (57.14%)	5 (35.71%)	1 (7.14%)	NR
Boeken et al. [25]	Small bead	63 ± 10 (43–81)	31	23	31 (57%)	18 (33%)	5 (9%)	NR
	Large bead	66 ± 9 (50–86)	22	8	22 (73%)	8 (27%)	0 (0%)	NR

Outcome Measures

The primary endpoint was overall survival benefit after DEBIRI treatment. The secondary endpoint was progression-free survival (PFS) and treatment responses based on Response Evaluation Criteria in Solid Tumours (RECIST) and Modified Response Evaluation Criteria in Solid Tumours (m-RECIST) criteria. Overall survival (OS) was the time elapsed between the treatment start date and death. PFS was taken as the time between the start of treatment and objective disease progression. The toxicity profile was based on adverse events (AE) occurring within 30 days of treatment. Most studies used the Cancer Therapy Evaluation Program's Common Terminology Criteria (CTCAE) for recording them.

Study Quality Assessment

Two independent reviewers assigned the remaining full-text articles' level of evidence. Quality assessment and bias risk were assessed using the tools depending on the type of study. For randomized controlled trials, the Cochrane Collaboration Risk of Bias Tool (CCRBT) was used; for cohort studies, the Newcastle-Ottawa Scale (NOS) was used; systematic reviews and meta-analyses were assessed with the Assessment of Multiple Systematic Reviews 2 (AMSTAR 2); and for narrative reviews, the Scale for the Assessment of Narrative Review Articles 2 (SANRA 2) was used. The accepted score for each assessment tool was 70%.

Data Analysis

Finalized articles fulfilling our inclusion criteria were listed, and the level of evidence for each of those publications was extracted. Finally, the results were tabulated and analysed using a qualitative approach addressing the following three categories: quality of studies (level of evidence), the number of studies (the number of published studies with the same patient cohort and study objectives), and consistency of results across studies (different studies concluded same results).

Results

Out of seven studies considered for final analysis, we found a pooled cohort of 302 patients. The mean age of the patients was 61.2 years ranging from 40.7 to 84 years. Male patients in our pooled cohort were 190 and females were 112. Six studies mentioned the Eastern Cooperative Oncology Group (ECOG) performance status. The majority of the patients had a performance status (PS) of zero (n = 111). The most commonly used DEBIRI beads were M1 (70-150 um) and M2 (100-300 um) but two studies reported the use of 40 um as well. The total number of DEBIRI treatments performed in our pooled cohort was 904. The median dose delivered was 100 mg, with a range of 50-200 mg. DEBIRI treatment characteristics are summarized in Table 4.

**Table 4:** DEBIRI treatment characteristics DEBIRI: drug-eluting beads, irinotecan.

Study	Number of treatments	Bead size	Dose delivered per session	Technical success
Mauri et al. [19]	80	40 μm	67.3 ± 28.1 mg/mL (range: 5.0–100.0 mg)	100%
Scevola et al. [20]	192	75-300 μm	200 mg	100%
Ngo et al. [21]	125	70-300 μm	100 (range: 50–200)	99%
Mauri et al. [22]	197	40 μm	150 mg	100%
Kong et al. [23]	46	100-300 μm	100 mg	100%
Fereydooni et al. [24]	32	70–150 μm	100 mg	100%
Boeken et al. [25]	232	70-300 μm	100 mg	100%

Of the patients, 92 (six studies) had a tumour burden <25%, whereas 83 patients had a tumour burden between 25% and 50%, and 48 patients had a tumour burden of more than 50%. Of the patients, 91 had monolobar liver disease, whilst 127 had bilobar liver disease. Liver involvement has been described in Table 5, and previous treatments are illustrated in Table 6.

**Table 5:** Extent of liver disease KRAS: Kirsten rat sarcoma viral oncogene; NR: not recorded.

Study				Tumour burden							KRAS		Extrahepatic disease
	Unilobar		Bilobar	≤30%	≤60%	≤25%	>25-≤50%	>50%	Number of lesions	Total sum of all target lesions	Wild type	Mutated type	
Mauri et al. [19]	8		10			12	5	1	9.1 (1-30)	46.9 (6.5–154.5)			Lungs: 11 (61.1)
													Lymph nodes: 3 (16.6%)
													Peritoneum: 4 (22.2%)
Scevola et al. [20]	27		35			NR	NR	NR	3.6 (range: 3–9)	6.2 (range: 2.5–6.2)	NR	NR	Excluded from study
Ngo et al. [21]	26 (49%)		27 (51%)			32 (60%)	14 (26%)	7	NR	NR			Lungs: 20 (83%)
													Lymph nodes: 2 (8%)
													Peritoneum: 3 (13%)
Mauri et al. [22]	21 (38%)		34 (62%)			9 (16%)	29 (53%)	17 (31%)	NR	42.0 ± 24.1	32 (58%)	23 (42%)	Lungs: 13 (43%)
													Lymph nodes: 1 (3%)
													Peritoneum: 3 (10%)
Kong et al. [23]	5 (31.3%)		11 (68.7%)	4 (25%)	12 (75%)				0-10 = 7 (50%) >10 = 7 (50%)	NR	11 (68.7%)	5 (31.3%)	Excluded from study
Fereydooni et al. [24]	4 (28.57%)		10 (71.43%)			13 (92.86%)	1 (7.14%)		NR	NR			4 (28.57%) - lung, lymph nodes, peritoneal
Boeken et al. [25]	Small bead	NR				16 (30%)	24 (44%)	14 (26%)	NR	NR	31 (57%)	23 (43%)	Lungs: 11
													Lymph nodes: 0
													Peritoneum: 1
	Large bead	NR				10 (33%)	10 (33%)	10 (33%)	NR	NR	15 (50%)	15 (50%)	Lungs: 1
													Lymph node: 0
													Peritoneum: 0

**Table 6:** Previous treatments NR: not recorded; MWA: microwave ablation; RFA: radiofrequency ablation; IRE: irreversible electroporation; SIRT: selective internal radiation therapy.

Study		Previous chemotherapy	Hepatic resection	Locoregional therapies
Mauri et al. [19]		3 lines	16 (88.9%)	NR
Scevola et al. [20]		2 lines	NR	NR
Ngo et al. [21]		2 (0-5)	21 (40%)	MWA – 4 (8%), RFA – 15 (28%), IRE – 3 (6%), SIRT – 1 (2%)
Mauri et al. [22]		>2	NR	NR
Kong et al. [23]		2 lines (at least 6 cycles)	NR	0 (0%)
Fereydooni et al. [24]		>2 11 (78.57%)/3 (21.43%)	NR	NR
Boeken et al. [25]	Small bead	3 (2-6)	NR	NR
	Large bead	3 (2–5)	NR	NR

The majority of the studies reported only grade 1 (G1) or grade 2 (G2) toxicities among the patients, with maximal toxicity of grade 4 (G4) in a few selected patients. Most studies did not report a reduction in carcinoembryonic antigen (CEA) levels; however, two studies mentioned a 50% reduction in CEA levels after DEBIRI. The response criteria that was unanimously used was RECIST. Complete response was reported in 23 patients, while partial response was documented for 21 patients. We found 51 patients to have stable disease following DEBIRI. Unfortunately, eight patients showed disease progression in our pooled cohort (four studies). The median overall survival in our pooled cohort was 19.52 months. The median PFS in our data was 5.76 months ± 1.56 months. DEBIRI efficacy and DEBIRI toxicity profile have been shown in Tables 7, 8.

**Table 7:** Efficacy of DEBIRI CR: complete response; PR: partial response; SD: stable disease; PD: progressive disease; NR: not recorded; RECIST: Response Evaluation Criteria in Solid Tumors; mRECIST: modified Response Evaluation Criteria in Solid Tumors.

Study		Response criteria	CR	PR	SD	PD	Median overall survival -months	Median progression-free survival - months	30-day mortality
Mauri et al. [19]		RECIST	16/18 (88.9%)	7/17 (41.2%)	3/17 (17.6%)	2	13.52	5.9	NR
Scevola et al. [20]		RECIST 1.1	7		16		51 months (n = 17)		0
Ngo et al. [21]							14.5 months (0.6–107)	5 (0.2–87)	NR
Mauri et al. [22]				1/55 (2%)	21/55 (38%)	1/55 (2%)	9.9 (95% CI: 6.2–14.2)	3.2 (95% CI: 3–4.1 months)	NR
Kong et al. [23]		m-RECIST	0	13 (81.2%)	2 (12.5%)	1 (6.2%)			0
Fereydooni et al. [24]		m-RECIST	0	0 (0%)	9 (69.23%)	4 (30.77%)	18.1 months		0
Boeken et al. [25]	Small bead	RECIST	0				15.59	7.55 months	0
	Large bead	RECIST	0				13.04	7.15 months	0

**Table 8:** Toxicity profile of DEBIRI DEBIRI: Drug-eluting bead, irinotecan; CTCAE: Common Terminology Criteria for Adverse Events; G: grade.

Study		Toxicity criteria	Overall toxicity	High-grade toxicity	Total adverse effects	Abdominal pain	Nausea & vomiting	Increase in transaminases during 3 days of the procedure	Cholecystitis
Mauri et al. [19]					39	10	6	0	
Scevola et al. [20]							30	15	
Ngo et al. [21]		CTCAE							1
Mauri et al. [22]		CTCAE	G1	None	30/197 (15%)	17/30 (57%)	41 (89.1%)/39 (84.8%)		
Kong et al. [23]		CTCAE	G1/G2	G3		35 (76.1%)			1 (2.2%)
Fereydooni et al. [24]		CTCAE		G4		7 (50%)	1 (7.14%)	2 (14.29%)	
Boeken et al. [25]	Small bead	CTCAE	G1/G2	G4	441	8 (2–10)			2 (2%)
	Large bead		G1/G2	G4	392	8 (4–10)			0

Discussion

Colorectal liver metastasis (CRLM) has always been a challenging disease to treat, but the past decade has shown that DEBIRI can be incorporated into mainstream treatment strategies. Our systematic review comes five years after the review published by Akinwande et al. [26]. Our systematic review indicates a definitive benefit for DEBIRI with acceptable toxicity profiles. We also found a very substantial technical success rate of chemoembolization from all the studies, which leads us to think about when it should be done as the only factor determining its initiation. The European Society of Medical Oncology and the National Cancer Network have given a criterion for the use of DEBIRI [27]; they are yet to add it to the standard treatment algorithm, which is the reason why in all the studies we included in the review showed at least two or three chemotherapy treatment lines completed before DEBIRI was advised. The survival outcomes we have gained from combining the studies warrant us to pose a question as to whether we need to initiate DEBIRI slightly earlier in the disease course along with systemic chemotherapy. Di Noia et al. in 2019 published a trial of DEBIRI with capecitabine showing relatively good outcomes in previously heavily treated patients [28]. Another attempt was made in 2020 to combine DEBIRI with mFOLFOX6 to attain a median PFS of at least nine months; however, despite being unable to achieve that, they did have a prolonged median overall survival of 37.4 months; the study also showed that grade 3 (G3) to grade 5 (G5) toxicities were reported when both lobes were treated at the same time [29]. One of the potential drawbacks associated with DEBIRI that may have been a limiting factor is its potential to induce hypoxic necrosis into tissues that will ultimately lead to increased expression of vascular endothelial growth factor (VEGF) in the tissues, hence further augmenting local relapse [30]. To address this, Fiorentini et al. (2020) published preliminary results of an ongoing randomized control trial comparing DEBIRI alone versus DEBIRI with bevacizumab. The initial results show that the latter seems tolerable and feasible, but long-term survival and PFS results are still awaited [31]. Nevertheless, DEBIRI alone in itself has a significant impact, as our review showed a median OS of 19.5 months; the median OS survival for refractory colorectal liver metastases has been reported to be around five months at best [32]. Our review findings are incongruent with a systematic review performed by Richardson et al., who concluded that DEBIRI had better outcomes concerning the quality of life and PFS when compared to irinotecan-based systemic chemotherapies [33].

The survival estimates can be imprecise due to underpowering sample sizes, PFS, and OS in many of the studies of DEBIRI. However, it is of note that it is challenging to acquire trials of near-perfect design, especially when the patient population is under investigation at multiple platforms for various other therapies showing promise. Nevertheless, there is enough evidence for continuing research and innovation for DEBIRI. One of the National Health Service (NHS) trusts has already formed an international registry named DLivERDEBIRI. This will also help gather a larger evidence body and provide valuable insight into long-term benefits.

Our systematic review highlights the potential of DEBIRI as a treatment for colorectal liver metastases. Our analysis provides substantial evidence supporting its efficacy and acceptable toxicity profiles. However, it is important to note that further investigation is required to understand the response of specific subtypes of colorectal cancer such as poorly differentiated and poor prognostic types. Addressing these subsets of patients is crucial for optimizing treatment strategies and tailoring therapies to individual needs. Future research should focus on conducting in-depth studies targeting these colorectal cancer subtypes and exploring the nuanced interactions between DEBIRI and tumour characteristics. Additionally, efforts should be directed towards enhancing the precision of patient selection criteria to maximize the benefits of DEBIRI for a broader spectrum of colorectal cancer patients. These advancements will significantly contribute to the evolving landscape of colorectal cancer treatment, paving the way for more personalized and effective therapeutic approaches.

Our review has many limitations, the greatest of which is the lack of substantial levels of evidence for the studies included, adding inherent bias risk to the review. Furthermore, we only included single-arm studies; hence a comparative analysis with other potential treatment strategies was not done. Another potential drawback could be the missing data due to most studies being case series and a lack of uniformity in reporting the treatment outcomes. Despite all these limitations, we firmly believe that we have gathered enough new evidence to pave the way for initiating randomized control trials that will give a clear picture of the actual overall effect of DEBIRI on refractory CRLM compared to systemic chemotherapy.

## Conclusions

Our review suggests that DEBIRI is a very useful treatment modality with an acceptable toxicity profile and a good median overall survival benefit for refractory colorectal liver metastasis. Although the highest level of toxicity recorded is G4 but most common is G1/G2 making this profile acceptable. Our review has taken into consideration mostly case series or single arms from the clinical trials. Given the nature of the topic and the treatment, the literature on the cohort we selected is limited. Further studies should be done to inculcate DEBIRI into the standard treatment algorithm for colorectal liver metastasis.
